# Supplementary material for: The Effect of Blood Contained in the Samples on the Metabolomic Profile of Mouse Brain Tissue: A Study by NMR Spectroscopy
Source: Molecules. 2021 May 22;26(11):3096. doi: 10.3390/molecules26113096 (PMC8196876; doi:10.3390/molecules26113096)
Supplement: Supplementary file 1 [file molecules-26-03096-s001.zip › molecules-1208481-supplementary.pdf]

Table S1. Chemical shifts of NMR signals used for metabolite quantification, and indication of metabolites those assignments was validated by spiking with standards.

	Group, multiplicity	Chemical shift, ppm	Verification by authentic compound	Relaxation time T_1, s
2-hydroxy-butyrate	CH_3, t	0.886	+	
Alanine	CH_3, d	1.470		3.1
AMP	CH, s	8.597	+	
Ascorbate	CH_2, d	4.501		
Aspartate	CH_2, dd	~2.8	+	
Choline	$(CH_3)_3$, s	3.189		2.6
Creatine	$(CH)_3$, s	3.920	+	3.3
Formate	CH, s	8.446		
Fumarate	$(CH)_2$, s	6.504		
GABA	CH_2, t	3.001	+	
Glutamate	CH_2, o	2.342	+	
Glutamine	CH_2, q	2.441	+	
Glycine	CH_2, s	3.548		
GSH	CH, dd	4.56	+	
Guanosine	CH, s	7.987	+	
Histidine	CH, s	7.060		6.9
Hypoxanthine	CH, s	8.175	+	
Inosine	CH, s	8.330	+	
Isoleucine	CH_3, d	1.003		
Lactate	CH, q	4.094		4.5
Leucine	$(CH_3)_2$, dd	0.954		2.2
myo-Inositol	CH, t	4.045		
N-Acetyl-Aspartate	CH_3, s	2.005		
NAD	CH, s	9.328		
Nicotinamide	CH, dd	8.926		
Phenylalanine	$(CH)_2$, tt	7.42		3.6
Phosphocholine	$(CH_3)_3$, s	3.208	+	
Phosphoethanolamine	CH_2, m	3.205		
Pyruvate	CH_3, s	2.362	+	
scillo-Inositol	$(CH)_6$, s	3.332	+	
Serine	CH, dd	3.826	+	
Succinate	$(CH_2)_2$, s	2.395		
Taurine	CH_2, t	3.412		2.8
Uracyl	CH, d	5.788		
Uridine	CH, dd	7.860	+	
Valine	CH_3, d	0.978		1.3

List of abbreviations: AMP – Adenosine monophosphate, GABA – γ-Aminobutyrate, GSH – Glutathione reduced, NAD - Nicotinamide adenine dinucleotide.
